# MicroRNAs in *Taenia solium* Neurocysticercosis: Insights as Promising Agents in Host-Parasite Interaction and Their Potential as Biomarkers

**DOI:** 10.3389/fmicb.2017.01905

**Published:** 2017-09-29

**Authors:** Renzo Gutierrez-Loli, Miguel A. Orrego, Oscar G. Sevillano-Quispe, Luis Herrera-Arrasco, Cristina Guerra-Giraldez

**Affiliations:** Neurocysticercosis Lab, Laboratorios de Investigación y Desarrollo, Facultad de Ciencias y Filosofía, Universidad Peruana Cayetano Heredia, Lima, Peru

**Keywords:** miRNAs, *Taenia solium*, neurocysticercosis, host-parasite interactions, biomarkers

## Abstract

MicroRNAs (miRNAs) are short, endogenous, non-coding, single-stranded RNAs involved in post-transcriptional gene regulation. Although, several miRNAs have been identified in parasitic helminths, there is little information about their identification and function in *Taenia*. Furthermore, the impact of miRNAs in neurocysticercosis, the brain infection caused by larvae of *Taenia solium* is still unknown. During chronic infection, *T. solium* may activate numerous mechanisms aimed to modulate host immune responses. Helminthic miRNAs might also have effects on host mRNA expression and thus play an important role regulating host-parasite interactions. Also, the diagnosis of this disease is difficult and it usually requires neuroimaging and confirmatory serology. Since miRNAs are stable when released, they can be detected in body fluids and therefore have potential to diagnose infection, determine parasite burden, and ascertain effectiveness of treatment or disease progression, for instance. This review discusses the potential roles of miRNAs in *T. solium* infection, including regulation of host-parasite relationships and their eventual use as diagnostic or disease biomarkers. Additionally, we summarize the bioinformatics resources available for identification of *T. solium* miRNAs and prediction of their targets.

## Introduction

Over the last decade, miRNAs have emerged as important factors in several biological processes, functioning as powerful post-transcriptional regulators. These small non-coding RNA molecules have the ability to bind complementary sequences in transcripts causing specific mRNA cleavage or translational repression (Jonas and Izaurralde, 2015).

MiRNAs secreted by parasitic helminths and detected in their hosts' blood represent promising candidates as biomarkers to diagnose infection (Cheng et al., 2013; Hoy et al., 2014; Tritten et al., 2014; Quintana et al., 2015). Besides, a role in host-parasite interactions, specifically in immune modulation, has been strongly suggested for miRNAs secreted by the nematode *Heligmosomoides* (Buck et al., 2014) and the trematodes *Fasciola* (Fromm et al., 2017) and *Schistosoma* (Zhu et al., 2016). Recently, miRNA screenings for several parasitic helminths have been completed providing new information into the fundamentals of helminth biology and pathogenesis (Cai et al., 2016). However, only limited and fragmented data are available for tapeworms, including *Taenia solium*.

Infection with *T. solium* is endemic in developing countries where free roaming pigs, the intermediate hosts, have access to human waste. However, cases in high-income countries are increasing because of migration of tapeworms carriers, who release infectious ova into the environment. Only humans can serve as the definitive host and carry the adult tapeworm in the small intestine, but they can also act as intermediate hosts and be infected with larvae, the stage that causes cysticercosis. In this disease, the larvae of the parasite, also known as a metacestode or cyst, develop from a blood-borne oncosphere (embryos liberated from eggs found in infected human feces) and lodge in muscles and/or brain (Garcia et al., 2003, 2014).

The brain infection known as neurocysticercosis (NCC) has heterogeneous symptoms that vary depending on the viability, location and number of cysts, and on the associated inflammation of the brain tissue. The most important clinical manifestations are seizures and epilepsy. NCC is thus a frequent cause of adult acquired epilepsy in low-income countries and is associated with high morbidity (Garcia et al., 2003, 2014; Nash and Garcia, 2011).

The chronic nature of the infection in NCC implicates a wide variety of host and parasite biological processes and interactions. Manifestations of disease occur months or decades after exposure, usually as a result of degeneration of parenchymal cysts and the resulting inflammatory responses. Until degeneration occurs, there is scarce or no effective inflammatory response to the cyst, as seen extensively in the brains of naturally infected pigs (Sikasunge et al., 2009). The parasite actively prevents this inflammatory response (Fleury et al., 2016), which prolongs its survival in the host. In humans, the appearance of symptoms is related mostly to perilesional oedema due to inflammation associated with degenerating cysts (Fujita et al., 2013; Garcia et al., 2014; Nash et al., 2017).

*T. solium*-derived factors have been implicated in this modulation; the intimate association between host and parasite and the increasing evidence that the immune response is highly controlled at the post transcriptional level (Davidson-Moncada et al., 2010), together with the evidence about miRNAs in other infections (Arora et al., 2017b), make miRNA-based mechanisms attractive for exploration in this disease. Importantly, larvae from *T. crassiceps* and *Mesocestoides corti*, commonly used to model NCC, have been reported to release miRNA-containing vesicles *in vitro*, which could come in contact with a host and be part of a communication mechanism (Ancarola et al., 2017).

Here we summarize the biology of NCC in which miRNAs could play a role as local regulators. Many observations about NCC rely on experimental data obtained from pigs naturally infected with *T. solium*, as this system has aided in the description of several biological events in the pathology of NCC. The recent findings of miRNAs in related parasites may motivate the exploration of the transcriptional mechanisms involved in NCC. We hypothesize that miRNAs identified in other cestodes might share functional features with those of *T. solium*. Currently, the genome of this parasite is being explored in the search for miRNAs (Pérez et al., 2017).

## miRNA biogenesis and its role in helminth-induced immune modulation

Biogenesis of miRNAs follows a canonical process that takes place between the nucleus and cytoplasm in the cell. In eukaryotes, miRNAs precursor genes reside in the genome, frequently as part of intergenic regions and introns of protein-coding genes (Lau et al., 2001). The biogenesis of these molecules starts with transcription by RNA polymerase II or III in the nucleus (Lee et al., 2004; Borchert et al., 2006). The long primary transcript or pri-miRNA features a hairpin secondary structure where the miRNA is located (Ha and Kim, 2014). A double stranded RNA-specific RNase, Drosha, cleaves the pri-miRNA, resulting in a small hairpin structure known as pre-microRNA, which is exported to the cytoplasm (Yi et al., 2003; Winter et al., 2009). Finally, the pre-miRNA is processed one more time by the RNase Dicer, producing a short unstable miRNA-miRNA duplex (Winter et al., 2009; Ha and Kim, 2014). This mature miRNA duplex is then loaded onto an Argonaute protein that removes one of the strands. The RNA-induced silencing complex (RISC) is finally assembled and ready to be functional as a gene silencing machinery (Winter et al., 2009).

Increasing evidence associates miRNAs to several events in immune response against helminths, like homeostasis, activation, cell differentiation and survival in both innate and adaptive immune species (Mehta and Baltimore, 2016; Arora et al., 2017b; Entwistle and Wilson, 2017). For example, macrophages are critical in defense against intestinal helminths (Anthony et al., 2006) and their activation, mediated by IL-4/IL-13 pathways, is known to be tightly regulated by expression of miR-342 (Czimmerer et al., 2016). Similarly, expression of miR-155 is essential for maturation of both T and B cells, and deficiency of this trait is associated with increased propensity for Th2 rather than Th1 responses (Rodriguez et al., 2007).

In contrast, parasite-induced dysregulation of host immune miRNAs is studied in a few parasites, and it might represent a valuable source of biomarkers to monitor disease progression or verify effectiveness of treatment. Remarkably, during active infection by *Schistosoma japonicum*, the immune cell-specific miRNAs 146a/b, miR-155, and miR-223 showed significant changes in their expression (Cai et al., 2013).

Based on these reports, it is possible that the immune response in NCC shares hallmarks found in other events governed by expression of miRNAs.

## NCC pathogenesis: biological events might be regulated by miRNAs

The study of NCC pathogenesis faces several limitations since the whole life cycle of *T. solium* is practically impossible to reproduce in a laboratory. Although different experimental animal models exist (Arora et al., 2017a), infected pigs, the natural intermediary host for *T. solium*, develop brain granulomas comparable to those observed in human NCC (Alvarez et al., 2002; Fleury et al., 2016). The study of naturally infected pig brains is therefore providing useful information about the biology of the host-parasite interaction in NCC (Arora et al., 2017a). Reports at the cellular and molecular level include the regulation of cytokines (Mahanty et al., 2015) and adhesion molecules (Singh et al., 2017), as well as the use of MRI to follow up cyst damage after treatment (Cangalaya et al., 2017).

After ingestion, the *T. solium* oncosphere migrates via the bloodstream and if it avoids destruction it will develop into a viable cyst (Garcia et al., 2003). Oncospheres lodge in small arterioles, which become enlarged and modified to nurture the parasite. Once in the brain, the viable cyst elicits none or very low inflammation in the surrounding tissues, remaining undetectable to the host immune system (Sikasunge et al., 2009). This observation has for long time suggested the existence of parasite-derived attenuating or modulating mechanisms, like the synthesis of proteins similar to those of the host (Flisser et al., 1986; White et al., 1997). Products secreted by the cyst have been shown to limit the viability of lymphocytes *in vitro* (Molinari et al., 2000), induce their apoptosis (Solano et al., 2006), and inhibit the expression of inflammatory cytokines (Arechavaleta et al., 1998). All these properties were lost when cyst secretion products were treated with RNases (Tato et al., 1995). This evidence supports the hypothesis of the RNA nature of the parasitic immunomodulatory agents and suggests transcriptional regulation by the parasite. However, the regulatory machinery remains to be described.

Studies in naturally infected pigs revealed extensive astrogliosis around *T. solium* cysts, suggesting the participation of glial cells in the activation of a following immune response (Sikasunge et al., 2009). Several miRNAs seem to be involved in the regulation of glial cells. For example, miR-124 was associated to microglial quiescence and modulation of monocyte and macrophage activation (Ponomarev et al., 2011). Similarly, miR-146 plays several roles in astrocyte-mediated inflammation by affecting the release of cytokines like IL-6 and TNF-α. This supports an important role of miR-146a in human neurological disorders associated with chronic inflammation (Iyer et al., 2012).

Degenerated and dead cysts are surrounded by inflammatory cells that infiltrate from the periphery into the central nervous system (CNS). However, how and why degeneration and inflammation occurs to and around cysts is unknown. This process is accompanied by disruption of the blood brain barrier (BBB) and increased vascular permeability, which allows for the infiltration of immune elements in inflammation, as seen in porcine NCC (Guerra-Giraldez et al., 2013). Recently the up-regulation of miR-150 was found to be associated to disruption and increased permeability of the BBB in a rat model of stroke (Fang et al., 2016), supporting a role for miRNAs in the dynamics of maintaining the BBB.

As to the role of the BBB in (porcine) NCC, our group has reported the upregulation of proinflammatory genes, like TNF-α, in pericystic brain tissue with vascular leakage (Mahanty et al., 2015). However, there is no documented evidence of miRNA mechanisms underlying this event. Promisingly, miR-146 was found to regulate inflammation in the CNS via NF-κB signaling pathways and expression of TNF-α during brain infection in mice by the nematode *Angiostrongylus cantonensis* (Yu et al., 2014a; Mo et al., 2016). Additionally, higher expression of miR-155, miR-206, miR-223, and miR-511 have been associated with progression of meningitis in the same mouse model (Yu et al., 2014b). These findings suggest the potential use of some miRNAs as indicators of disease severity in the brain.

Furthermore, our group also reported that BBB disruption in porcine NCC is also found in post-treatment inflammation (Guerra-Giraldez et al., 2013). For this reason, the recognition of miRNAs associated to changes in the BBB permeability would be promising elements for ascertaining effectiveness of treatment or disease progression.

## miRNAs in *Taenia solium*: molecular signatures shared with other cestodes

To this date, miRNAs have been explored in only a few platyhelminthes, notably *Echinococcus* (Jin et al., 2013). Only limited and fragmented data are available for other tapeworms. Although, there is very little information about miRNA diversity and functional approaches in *T. solium*, some studies made in other species from the genus *Taenia* and related organisms could provide valuable information for the prediction of homologous and species-specific miRNAs in *T. solium*.

Despite the fact that miRNAs are essentially found within the cell, some have been detected in the extracellular environment, most likely being secreted for cellular communication. (Manzano-Román and Siles-Lucas, 2012) Moreover, the accumulating evidence that miRNAs are released into circulation and their ease of detection using quantitative methods such as qPCR, have positioned these molecules as novel non-invasive biomarkers (Weiland et al., 2012; Hoy et al., 2014).

Screenings for miRNAs in *Echinococcus multilocularis, E. granulosus*, and *Taenia multiceps* have been published (Wu et al., 2013; Bai et al., 2014; Cucher et al., 2015). These data represent a good resource to carry out comparative comprehensive analyses with other cestodes.

In addition, high-throughput analysis in *Taenia ovis* metacestodes have led to the identification of 33 known miRNA sequences and the recognition of the miR-71/2b/2c cluster, which is conserved in six other cestodes including *T. solium* (Zheng, 2017). In *E. multilocularis*, miR-71 was found to modulate nitric oxide production in macrophages, suggesting a role in host-parasite interactions (Zheng et al., 2016). Similarly, nematode-derived miR-71 was detected in the plasma or serum from individuals infected with *Onchocerca volvulus* (Quintana et al., 2015). These evidences suggest the potential use of miR-71 as a biomarker for diagnosis.

A comparative analysis of the miRNAs profile between *T. saginata* and *T. solium* adult worms revealed a high similarity between these two species, most surely due to their close identity, with predominant expression of miR-7-5p, miR-71, miR-277, miR-219-5p, and miR-2b-3p. Of note, miR-10-5p was a read unique for *T. solium* (Lin et al., 2014). We can elaborate that this reflects differences in physiology or life cycle.

Although, no functional tests were performed, the deep sequencing of plasma from *S. japonicum*-infected rabbits and human patients led to the identification of miR-10 and miR-277, respectively (Cheng et al., 2013; Hoy et al., 2014), suggesting their applicability as parasite biomarkers.

In addition, the finding of miRNAs in blood even when the parasite does no reside directly in the blood stream, like with *Schistosoma*, (Cai et al., 2016) indicates miRNA are robust and may affect body processes far removed from their site of release.

## Bioinfomatic tools for miRNA identification and prediction of targets

High-throughput sequencing from small RNA libraries represents a breakthrough for *de novo* identification of miRNAs and further experimental recognition. Nevertheless, this approach is not always easily affordable, especially for research groups in endemic regions where facilities are limited.

In this scenario, bioinformatics is an invaluable resource in the identification of new microRNAs. The methods for computational prediction of potential miRNA sequences are increasingly more sophisticated and support the possible use of the genomic data available from *T. solium* to perform homologous mapping based on annotated helminthic miRNA populations.

To this date, *miRBase* (http://www.mirbase.org/search.shtml) is probably the most complete miRNAs database. It brings together open-access information about reads and annotated sequences of miRNAs and represents a first step in homologous identification. Interestingly, *miRNEST* database (http://mirnest.amu.edu.pl) offers six records available for *T. solium* from which is possible to address, although limited, an identification based on homology. After looking for coincidences, complementary analyses based on structural features of miRNAs should be performed.

One important feature of miRNAs is their phylogenetic conservation (Zhang et al., 2006; Li et al., 2010), which is why alignment tools like *BLAST* (Camacho et al., 2009), although not perfect (Legendre et al., 2005), are relevant when performing prediction of miRNA homologs through sequence similarity (Wang et al., 2005; Dezulian et al., 2006; Kim et al., 2006). *deepBlockAlign* (http://rth.dk/resources/dba/) is a more potent alignment tool, created specifically for small RNA sequences (Pundhir and Gorodkin, 2013).

Moreover, the thermodynamic stability of the secondary structure of precursor miRNAs is evidenced in their low minimum free energy of folding (Bonnet et al., 2004). Based on this, elaborate strategies involve machine-learning algorithms. For example, *HuntMi* (http://adaa.polsl.pl/agudys/huntmi/huntmi.htm) is an algorithm that is constantly updated with several miRNA parameters from thousands of reported miRNAs, so that the program keeps “learning” what defines a miRNA and looks for the best coincidences (Gudyś et al., 2013).

To further characterize predicted or already reported miRNA sequences in a functional way, target prediction is a step that complements and can be done prior to experimental validation. Tools like *RNAhybrid* (https://bibiserv.cebitec.uni-bielefeld.de/rnahybrid) use the minimum free energy of base pairing between miRNA and a target (Krüger and Rehmsmeier, 2006). Similarly, *PicTar* (http://www.pictar.org/) uses annotated miRNA sets and multiple alignments of orthologous nucleotide sequences to generate gene scores by their likelihood of being targets.

Together, these resources provide useful strategies for miRNA prediction and target identification, which would precede experimental demonstration.

## Conclusions and perspectives

The complex disease progression of NCC involves a wide variety of biological processes that might be affected by miRNA regulation mechanisms. Since miRNA of parasitic helminths are receiving increasing attention as novel regulators in disease, the data available for miRNA helminths and the advantageous use of bioinformatics tools for *in silico* prediction are valuable resources in the search of still unknown *T. solium* species-specific miRNAs.

The study of miRNAs in *T. solium* is likely to add information about the organism and its interaction with the host as well a way to diagnose infections and manage the disease.

## Author contributions

RG, MO, and CG designed the outline of the mini review. RG, MO, OS, and LH collected information. RG wrote the first draft of the article. CG finished the critical revision of the article.

### Conflict of interest statement

The authors declare that the research was conducted in the absence of any commercial or financial relationships that could be construed as a potential conflict of interest.
